# Enhancing green total factor productivity through manufacturing output servitization: A case study in China

**DOI:** 10.1016/j.heliyon.2023.e23769

**Published:** 2023-12-17

**Authors:** Hongsen Wang, Martin Lockett, Dongni He, Yiqing Lv

**Affiliations:** a School of Business Administration, Northeastern University, Shenyang, 110167, China; b Nottingham University Business School China, University of Nottingham Ningbo, 315100, China; c The Guangzhou Institute of the Greater Bay Area, Guangzhou, 510700, China; d School of Economics, Sichuan University, Chengdu, 610065, China

**Keywords:** Manufacturing output servitization, Green total factor productivity, Sustainability, Environmental impacts, Economic efficiency

## Abstract

In the context of the growing environmental pollution and resource depletion caused by traditional manufacturing industries, the need for sustainable and eco-friendly practices has become a critical issue for the upgrading and transformation of the manufacturing industry worldwide. Based on data from listed manufacturing companies in China, which is the world's largest manufacturing country and exhibits significant diversity regarding the ownership, scale and level of enterprises, the impact of manufacturing output servitization on green total factor productivity (GTFP), which is a measurement of economic efficiency that takes into account environmental impacts, is analyzed in this article. The results show that manufacturing output servitization can improve the GTFP of enterprises, and this can be achieved through mechanisms such as increased profitability and innovation capabilities. The positive effect on the GTFP of enterprises in less developed regions is greater than that in developed regions and is more significant for private and foreign-funded enterprises than for state-owned enterprises. The companies that adhere to the Global Reporting Initiative framework for environmental, social and governance reporting experience a more significant positive impact on GTFP as a result of their manufacturing output servitization efforts. This research offers valuable insights into the potential of servitization as a strategy for enhancing GTFP and provides actionable guidance for policy-makers and industry stakeholders seeking to align manufacturing practices with sustainability goals.

## 1 Introduction

The manufacturing industry is an important pillar of China's economic growth. According to statistics from 2022, the manufacturing industry accounts for over 27 % of China's gross domestic product (GDP) [1]. However, the manufacturing industry also notably contributes to China's environmental challenges. Manufacturing energy consumption accounts for approximately 60 % of China's total, and carbon emissions account for approximately 50 % [1]. Therefore, addressing the environmental issues faced by China's manufacturing industry is imperative. Furthermore, as the world's largest manufacturer, China bears a significant industrial production footprint. Various indicators, such as Green Total Factor Productivity (GTFP), have been formulated to quantify the rapid environmental impact and assess the sustainability of economic sector development.

With the intensification of global climate change and China's strict commitment to environmental policies, the manufacturing industry urgently needs to transition to greener and more sustainable practices. However, the implementation of these energy-saving and emission-reducing policies also poses challenges. For example, manufacturing companies may struggle to maintain a competitive advantage while maintaining compliance with stringent environmental regulations. Therefore, finding a balance between economic growth and environmental sustainability is crucial for the country's long-term development.

To address this challenge, an increasing number of Chinese enterprises are turning to the concept of manufacturing servitization. This is a paradigm shift where traditional manufacturing companies strive to modernize their industrial structure by transitioning into service-oriented enterprises. Such a transition involves both input and output servitization. Input servitization refers to the increasing reliance of manufacturing companies on service elements in their production inputs. Output servitization refers to the provision of more added services by manufacturing companies through their products. Manufacturing servitization has the potential to drive industrial modernization by improving GTFP through the mechanisms of enhanced efficiency, adaptability, and profitability. It departs from the traditional sole focus on production and aims to address the environmental issues inherent to the traditional manufacturing model. This helps to tap into the potential for manufacturing industry development while reducing resource consumption. For example, some automobile manufacturers have started to offer car-sharing and leasing services, thereby reducing the use and waste of cars and minimizing environmental impact. In addition, some manufacturing companies have achieved steady profit growth and reduced resource waste by providing after-sales service, maintenance, and customized services.

**Fig. 1:**
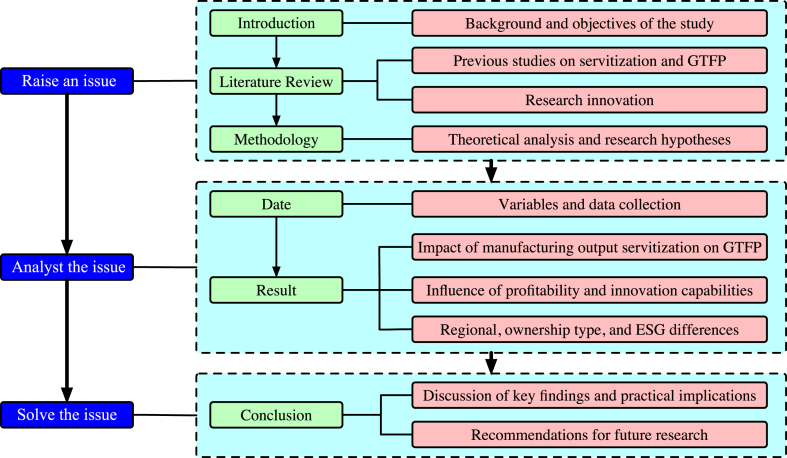
Flowchart of the research framework.

However, for manufacturing enterprises, the adoption of this model is hindered by path dependence, lack of government support and guidance, limited awareness of the benefits of servitization, and developmental constraints. As a result, the level of servitization in China's manufacturing industry currently lags behind the average level in developed countries [2]. In this article, the impact of manufacturing output servitization on GTFP in China's manufacturing industry is examined through the use of listed manufacturing companies as a case study. The mechanisms underlying the relationship between servitization and GTFP, including the roles of profitability and innovation capabilities, are explored. Furthermore, the heterogeneity of the impact of servitization on GTFP among the different enterprise types and regions in China. The aim of this research is to provide insights and recommendations for policy-makers and business leaders to support servitization as a means of achieving environmental, social, and economic goals both in China's manufacturing industry and in the global context. The research framework flowchart of this paper is depicted in Fig. 1.

## 2 Literature review

Manufacturing servitization is a process of adding services to manufacturing processes, including after-sales services, maintenance, and repair services, among others [3]. This process is aimed at increasing the value of products and providing a better customer experience [4]. Furthermore, a diversified and service-intensive industrial structure can better bolster economic resilience and facilitate sustained long-term growth [5], making manufacturing servitization a pivotal area for the transformation and upgrading of manufacturing [6]. It is also a form of organizational innovation that positively impacts production efficiency [7]. It brings about a synergistic effect between industries, enhancing the quality of economic growth through collaborative innovation between manufacturing and productive service sectors [8]. Additionally, it generates a competitive effect within the industry by offering opportunities for enterprises to enhance market competitiveness through proactive customer interaction [9]. Manufacturing servitization also affects internal resource allocation, improving production efficiency through enhanced research and development capabilities, high-level human capital attraction, and increased knowledge service elements [10]. Lastly, it drives Total Factor Productivity (TFP) growth by improving technological innovation capabilities and efficiency through product and customer-oriented services [11].

While most studies have demonstrated the considerable economic advantages of manufacturing servitization [12,13], some have highlighted the challenges and costs faced by manufacturing companies during the process of service-oriented transformation [14,15]. Moreover, the impact of service-oriented transformation on manufacturing enterprise performance may not be a simple linear relationship [16]. Xing et al. [17] explored the nonlinear correlation among digitalization, servitization transformation, and innovation performance involving 287 Chinese manufacturers. They showed that manufacturing servitization incurs challenges and costs. Their findings suggest that digitalization is not an independent prerequisite for manufacturers to achieve excellent innovation performance. Therefore, investigating the relationship between manufacturing servitization and economic benefits in a comprehensive and scientific manner is critical.

Early foreign scholars believed that strict resource constraints and environmental regulations were beneficial for improving the green total factor productivity and competitive advantage of enterprises, known as the “Porter hypothesis” [18]. Additionally, some studies have suggested that a shift toward service-oriented manufacturing (SOM) can also result in environmental benefits [19]. For example, Li et al. [20] combined the coverage of the World Input‒Output Database, China Industrial Enterprise Database, and China Industrial Enterprise Pollution Emission Database for the period from 2000 to 2012 and found that digital servitization has significantly contributed to reducing the pollution from Chinese manufacturing enterprises. However, other studies have indicated that this relationship is industry-specific and may even have a U-shaped or negative trend in regard to environmental benefits [21,22]. For example, Tang et al. [23] analyzed the multidimensional effects of input services on manufacturing carbon emissions using cross-national industrial group data collected from the global manufacturing industry between 2000 and 2014 and concluded that the carbon emission reduction effect of manufacturing service inputs is heterogeneous across different emission intensities. Therefore, it is important to examine the relationship between manufacturing servitization and environmental benefits and to identify the conditions under which environmental benefits are likely to be realized.

GTFP is an important measure of economic growth that accounts for environmental sustainability, which is defined as the ratio of output excluding nongreen products to all inputs [24]. The concept of GTFP is gaining popularity as a means of assessing the impact of economic growth on the environment [25]. Scholars have taken a multidimensional approach to comprehensively consider factors such as energy use, environmental impact and economic output and applied data envelopment analysis (DEA) to calculate comprehensive GTFP [26]. This illustrates that GTFP plays an important role in modern management as a measurement standard for balancing economic benefits and environmental costs. However, most researchers have mainly studied the impact of exogenous factors on GTFP, such as financial services [27], international direct investment [28] and environmental regulation policies [29]. Therefore, investigating the relationship between manufacturing firms’ output servitization and GTFP from an endogenous perspective and identifying the mechanisms underlying this relationship is necessary.

In summary, the previous literature has emphasized the importance of studying the relationship between manufacturing servitization and economic or environmental benefits, as well as the mechanisms underlying this relationship [30,31]. However, the implementation of manufacturing output servitization in many countries, including China, faces challenges that require the application of policy support, technological innovation, and collaboration among stakeholders [32]. Furthermore, balancing economic benefits with environmental benefits is extremely important. Specifically, there is a need to examine the impact of manufacturing firms’ output servitization on GTFP and to analyze the heterogeneity among different groups.

By analyzing data from listed manufacturing companies in China, the aim of this study is to investigate how servitization can contribute to improving economic efficiency while also reducing environmental impacts. Specifically, the impact of manufacturing output servitization on profitability and innovation capabilities is examined in this study, and the heterogeneity among manufacturing enterprises based on different ownership types (state-owned, private and foreign-funded), regions (developed, less developed), and environmental, social and governance (ESG) factors is analyzed. Through this research, we hope to fill gaps in the existing literature and chart a conceivable path toward a greener, more economically prosperous, and sustainable future for the global manufacturing industry.

## 3 Theory and hypothesis

### 3.1 Conceptual definition

Total output can be divided into desirable output and undesirable output, as shown in equation (1). Desirable output represents economic benefits, such as profits. Undesirable output represents environmental costs, such as carbon emissions. Both of these output types arise from the production process. The ratio of desirable output to undesirable output is influenced by the level of GTFP [33]. The higher the level of GTFP is, the higher the economic benefits and the lower the environmental costs. According to Equation (2), GTFP can be deduced from inputs (including labor, capital, energy, etc.) and expected outputs.

(1) Totaloutput=Desirableoutput+Undesirableoutput=Physicalproducts+Serviceproducts

(2) Totaloutput−Undesirableoutput=Desirableoutput=GTFP∙f(Labor,Capital,⋯)

Total output can also be divided into physical products and service products [34], as shown in equation (1). Physical products refer to traditional industrial manufactured goods. Service products refer to the presales and after-sales services attached to manufactured goods. The proportion of service product revenue to total revenue can be seen as a representation of the level of manufacturing output servitization [35]. It is important to note that the shift toward manufacturing output servitization does not include a complete replacement of physical products but rather the adding of value through the attachment of services to physical products.

Since service products have high value added, they can enhance a company's profitability while also improving levels of customer satisfaction and loyalty [36]. This can encourage innovation and technological advancements within the company, which may lead to an increase in GTFP levels. Moreover, manufacturing servitization can promote the synergistic development of the industry chain, thus contributing to the improved efficiency and competitiveness of the entire industry [37] and potentially raising the GTFP levels.

### 3.2 Theoretical analysis

Manufacturing output servitization refers to the business practice in which manufacturing enterprises not only engage in traditional product manufacturing but also provide value-added services such as design, development, logistics, and after-sales services. This emergent business approach is founded on service economics and value chain theory. In service economics, it is argued that services are the key drivers of economic growth in modern societies, while value chain theory stresses that enterprises can increase their profit margins by continuously incorporating additional value-added features [38,39]. Given these theoretical underpinnings, manufacturing output servitization has garnered attention as an innovative business model across both academia and industry.

In the context of manufacturing output servitization, enterprises offer comprehensive product solutions tailored to meet customer demands, encompassing everything from design and production to sales and after-sales services [40]. For instance, in the automotive manufacturing industry, manufacturers not only provide cars but also extend services such as loans, insurance, and maintenance. Similarly, in the machine tool manufacturing industry, manufacturers offer presale consultation, equipment installation and commissioning, and after-sales technical support. In some cases, manufacturers even alter their pricing and business models, such as charging a monthly fee for electric vehicle battery charging and replacement. The crux of SOM lies in the provision of technology, management, design, and other services to optimize enterprise resource allocation, enhance product quality, and minimize resource waste and environmental pollution. By adopting sustainable production practices, enterprises can reduce their reliance on natural resources, curb their waste emissions, and consequently improve their resource utilization efficiency while mitigating environmental pressures [41].

However, in-depth empirical research is needed to further investigate the impact of output servitization in the manufacturing industry on GTFP while taking into account other potential factors. Based on the above, *Hypothesis 1* is proposed.

H1 Manufacturing output servitization leads to an increase in GTFP.

### 3.3 Influence mechanisms

According to the literature review and theoretical analysis, the possible impact pathway of manufacturing output servitization on GTFP is as follows.

#### 3.3.1 Performance effect

Manufacturing output servitization has the potential to enhance competitiveness and profitability for enterprises. By prioritizing service quality and customer experience, manufacturing servitization enables enterprises to better cater to consumer needs. The service-oriented approach allows manufacturing enterprises to offer a wide range of value-added services, including customized solutions, data analysis, and remote monitoring [42]. As a result, the added value and market competitiveness of their products are augmented. Additionally, by offering green products and services, enterprises can improve the quality and performance of their offerings, leading to increased customer satisfaction and a stronger competitive edge.

Furthermore, manufacturing output servitization can contribute to increased output and revenue by improving profitability. Through the integration of production factors using scientific and technological advancements and information technology, manufacturing servitization facilitates optimal resource allocation [43]. Moreover, firms can enhance profitability by streamlining production processes, shortening logistics cycles, and reducing inventory. These measures not only lower production costs but also enhance production efficiency, resulting in a more energy-efficient, resource-efficient, and material-efficient production process. This ultimately leads to improved profitability and financial performance for the enterprise.

Consequently, Hypothesis 2 is proposed.

H2 Manufacturing output servitization enhances GTFP by improving profitability.

#### 3.3.2 Research and development effect

Manufacturing output servitization has the potential to improve research and development (R&D) within enterprises. This often involves the adoption of cutting-edge technology and management methodologies, such as artificial intelligence, the Internet of Things and big data analysis, to enhance technical proficiency and innovation capacity, and facilitating sustainable production practices [44]. These technological tools enable enterprises to more effectively manage and allocate resources, enhance green production, and reduce production costs while improving efficiency.

Additionally, manufacturing output servitization can contribute to the reduction of resource consumption and the promotion of clean production through enhanced R&D. By fostering enterprise innovation through technology spillovers and learning effects, manufacturing can elevate technical proficiency and improve production and energy efficiency. The service-oriented transformation can also enhance production, organizational, and management efficiency, optimize internal decision-making, and directly or indirectly reduce resource consumption during operations [45]. Furthermore, the separation of service and production functions can encourage firms to focus on knowledge-intensive activities and outsource resource-intensive tasks to other countries, leveraging their comparative advantages, optimizing resource allocation, and enhancing GTFP.

Consequently, Hypothesis 3 is proposed.

H3 Manufacturing output servitization enhances GTFP by augmenting firm innovation capabilities.

**Fig. 2:**
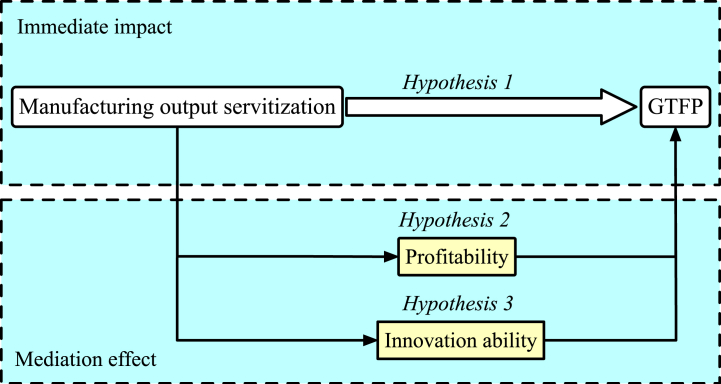
Analysis of the influence path.

In summary, the influence path is illustrated in Fig. 2.

## 4 Model and variable description

### 4.1 Model setup

In this paper, the ordinary least squares (OLS) method is used to examine the impact of manufacturing servitization on GTFP, as shown in Equation (3):

(3) $$\begin{array}{c} {\ln\;GTFP}_{it}=\beta_{0}+\beta_{1}{\ln\;Ser}_{it}+\beta_{2}{Control}_{it}+\gamma_{i}+\delta_{t}+\varepsilon_{it} \end{array}$$

where i and t denote enterprise and year, respectively. GTFP represents green total factor productivity, Ser denotes the level of manufacturing servitization, $\gamma_{i}$ and $\delta_{t}$ are the fixed effects of enterprise and year, $\varepsilon_{it}$ is the random disturbance term, β is the parameter to be estimated, and Control refers to the group of control variables.

### 4.2 Variable description

#### 4.2.1 Green total factor productivity

Drawing on the approach of Chen et al. [46], the DEA-SBM model is adopted in this study to calculate GTFP, which accounts for both desirable and undesirable outputs. It is presumed that each output unit in a firm is represented by two inputs (capital and labor), one desirable output (total revenue), and one undesirable output (carbon emissions). The formula for computing capital input is calculated by cash paid for operating leases, cash paid for acquisition and construction of fixed assets, intangible assets, and other long-term assets minus the net cash receipts from disposing of such assets. Labor input indicates the number of registered (employed) employees as reported in the company's annual report. Additionally, total revenue denotes the full sum of all revenues generated during business activities, while carbon emissions represent the comprehensive greenhouse gas emissions created by the company. The data on greenhouse gas emissions from listed companies are sourced from their annual reports, corporate social responsibility reports, sustainable development reports, etc. The data sources are authoritative and stable, primarily encompassing carbon dioxide, and they can provide a relatively comprehensive and objective presentation of the relevant environmental data of listed companies.

The choice to measure capital in monetary terms and labor in terms of the number of employees is based on practical considerations. Capital input is measured in monetary terms because doing so enables a comprehensive assessment of various types of assets, including operating leases, fixed assets, and intangible assets, which all have different monetary values. On the other hand, labor input is measured by the nominal employee headcount, as this number provides a readily available and easily comparable metric for assessing the amount of labor employed by a firm. To compute GTFP using the DEA-SBM model, both capital and labor inputs, total revenue and carbon emissions are utilized. The model accounts for the efficiency with which firms utilize their inputs (capital and labor) to generate outputs (revenue and carbon emissions). It used to assess the relative performance of each firm by comparing firm efficiency scores to that of the best-performing firm in the sample. This enables an evaluation of productivity growth that is adjusted for both desirable (revenue) and undesirable (carbon emissions) outputs.

#### 4.2.2 Manufacturing output servitization

To determine whether a company is involved in service businesses, a preliminary assessment based on business scope and other relevant information was conducted. For instance, when a listed manufacturing company primarily produces and sells automobile engines and components, castings, and offers automotive maintenance, machining, and technical consulting services, the company is noted as providing some services alongside its manufacturing goods, which exemplifies the concept of “production + service.” Furthermore, these company's annual reports were examined to validate their business descriptions and determine if they involved service industries. From the companies that disclose their service revenue, the proportion of service revenue to total revenue of the primary business was utilized to measure the degree of manufacturing output servitization.

#### 4.2.3 Control variables

(1) Enterprise growth capability (lnGro) is defined as the ratio of the owner's equity at the end of the period to that at the beginning of the period. Improving enterprise growth capability can foster investment and innovation in green production and is closely linked to GTFP. (2) Enterprise financial capability (lnFin) is expressed as the ratio of total liabilities to total assets. Greater financial support can augment enterprise income, bolster brand image, and encourage green production. (3) Capital intensity (lnCap) is defined as the ratio of fixed assets to total employees. High capital intensity implies that companies need more funds for equipment procurement, which may constrain their innovation and development in regard to environmental protection. (4) Enterprise age (lnAge) is represented by the number of years an enterprise has been in operation. In certain cases, young enterprises might place more emphasis on GTFP, particularly start-ups that are focused on environmental protection. (5) Enterprise size (lnSize), represented by the natural logarithm of enterprise revenue, may exhibit differences in production structure between large and small enterprises. (6) Energy intensity (lnElec) is calculated by the natural logarithm of electricity consumption per unit of regional (provincial-level) GDP, thus reflecting resource utilization efficiency in a region. The rational utilization of resources and the enhancement of resource utilization efficiency can reduce waste and environmental pollution.

#### 4.2.4 Mediating variables

According to the previous analysis, the following mediator variables are selected. (1) Enterprise profitability (Pro) is defined as the ratio of net profit to total asset balance. Manufacturing output servitization may enhance customer service quality and satisfaction, streamline production processes, and increase asset returns, enabling firms to allocate more resources to environmental protection and green production. (2) Enterprise innovation capability (Inn), represented by the proportion of research and development (R&D) investment to operating revenue, plays a crucial role in supporting corporate innovation. Manufacturing companies could accumulate a wealth of knowledge and experience through their service processes, thereby enhancing their innovation capability. Improving firm innovation capability may help companies enhance environmental technologies and optimize carbon management, thereby contributing to green production.

### 4.3 Data sources

This study utilizes data from listed manufacturing companies in China, where available, covering the period from 2012 to 2020. This is because the systematic and large-scale disclosure of carbon emissions data by Chinese listed companies only began after 2012, making it difficult to obtain data for the years prior to that point. The data are primarily taken from listed company data, environmental performance data and the thematic data of the China Economic and Financial Research Database. Companies that were bankrupt during the 2012–2020 period and those listed after 2018 were excluded to ensure that the sample companies had all maintained stable operations for at least three years. Companies operating for at least three years have a certain operational history, and their business and financial conditions are relatively stable. Companies listed for less than three years or those that went bankrupt midway were excluded because their shorter operational time or poor performance may not provide sufficient data support for the research and could also affect the stability of the sample data. Furthermore, since it was not mandatory to disclose corporate environmental information until 2022 and companies are not obligated to disclose revenue by income category, some manufacturing companies may lack data on carbon emissions or service revenue for certain years, resulting in incomplete data and a small sample size in this study. This study uses a sample of companies registered in 26 out of the 34 provincial-level administrative regions in China and covers 24 out of the 31 listed subindustries within the manufacturing sector. The data used in this study are based on unbalanced panel data. Descriptive statistics of the variables are presented in Table 1.

**Table 1:** Descriptive characteristics of the sample data.

Variables	Meaning	Observation	Mean	Standard Deviation	Min	Max
lnGTFP	Green Total Factor Productivity	106	0.041	0.224	−0.350	1.373
lnSer	Manufacturing output servitization	213	−0.038	0.081	−0.958	0.340
lnGro	Growth capability	212	0.135	0.231	−1.151	1.496
lnFin	Financial capability	213	−0.746	0.420	−2.724	0.102
lnCap	Capital intensity	213	12.815	0.876	9.439	14.717
lnAge	Enterprise age	213	3.113	0.250	1.946	3.526
lnSize	Enterprise size	213	23.654	1.612	18.444	27.441
lnElec	Energy intensity	213	−2.298	0.472	−3.142	−0.952
Pro	Enterprise profitability	213	0.057	0.069	−0.270	0.436
Inn	Enterprise innovation	200	4.485	4.731	0.002	57.310

The calculation of the maximum VIF value of the data variables as 1.12 excludes the influence of multicollinearity on the model regression. Subsequently, the result of the Breusch‒Pagan LM test indicates of the lack of cross-sectional dependence issues. In addition, the D-Fuller statistic is −7.765, which is less than −3.576 (left-tailed test), thus the hypothesis of “the existence of a unit root” for GTFP is rejected at the 1 % level. The P-Perron test also yielded consistent results. In summary, it is concluded that GTFP can be considered stationary.

## 5 Regression results

### 5.1 Benchmark regression

To analyze the relationship between manufacturing output servitization and GTFP, a fixed effect model based on the results of a Hausman test is utilized. The benchmark regression relies on OLS, and the results are presented in Table 2. Column (1) depicts the model without control variables or fixed effects. Column (2) presents the model incorporating fixed effects. Columns (3) and (4) display the model integrating both control variables and fixed effects. The findings indicate that regardless of whether fixed effects are included, manufacturing output servitization is always significantly and positively correlated with GTFP. Therefore, H1 is supported.

**Table 2:** Benchmark regression results.

Variables	(1)	(2)	(3)	(4)
lnSer	0.697*** (0.200)	0.794* (0.451)	0.821* (0.435)	1.011* (0.530)
lnGro			0.825** (0.392)	0.831** (0.391)
lnFin			0.845** (0.352)	0.802** (0.319)
lnCap			−0.408** (0.182)	−0.418** (0.177)
lnAge			0.256 (0.200)	1.067 (0.952)
lnSize			0.313 (0.235)	0.307 (0.230)
lnElec				−1.037 (0.952)
N	106	106	106	106
R^2^	0.105	0.708	0.791	0.820
Fixed effect	No	Yes	Yes	Yes

Note: *, **, and *** represent significance at the 10 %, 5 %, and 1 % levels, respectively. Robust standard errors are displayed in parentheses.

As shown in Table 2 (4), for every 1 % increase in manufacturing servitization, GTFP increases by 1.011 %. The reason behind this positive relationship may be twofold. First, manufacturing output servitization enables fine control and optimization of production processes through digital technology and informationization. This level of control promotes the reduction of resource waste, saves energy, and lowers emissions, thus achieving sustainable development and improving GTFP. This is in line with the findings of Wang and He [47], who concluded that manufacturing servitization can reduce carbon emissions. Second, as people's environmental awareness increases, the demand for environmentally friendly products grows accordingly. Manufacturing companies are transforming their value chain from being manufacturing centered to being services centered, thus optimizing production structure and product quality. This not only increases the added value of products but also reduces resource waste and environmental pollution. For example, Nica et al. [48] identified the role of services in enhancing sustainable industrial value within manufacturing systems. Thus, the output servitization helps to improve enterprise brand image and market competitiveness, thereby improving GTFP.

Regarding the control variables, the analysis reveals the following findings: (1) Enterprise growth capability is positively correlated with GTFP. Companies with strong growth capabilities invest more resources and technology in promoting innovation and technological upgrading, meeting their social responsibility, achieving a win‒win situation between economic and environmental benefits, and promoting the transformation of enterprises toward green production. (2) The enterprise debt ratio is positively correlated with GTFP. The asset-liability ratio reflects the proportion structure of internal and external financing of enterprises. Many studies have found that companies’ R&D activities primarily rely on internal financing [49]. However, a high debt ratio prompts companies to pay more attention to efficiency, risk management and sustainable development, which includes environmental protection and social responsibility. (3) Capital intensity is negatively correlated with GTFP. Industries that are capital-intensive require large amounts of capital and energy for production, which may result in more waste and pollutant emissions, thus adversely affecting the environment. They are also likely to be less agile in responding to changing demand given their high sunk investment costs in existing production-centric business models. (4) The relationship between enterprise age or size and GTFP is not significant. Therefore, both young and mature companies, as well as large and small enterprises, should realize the importance of GTFP for sustainable development, actively explore feasible green development paths, and take corresponding measures based on specific situations to promote the improvement of GTFP. (5) The negative correlation between energy intensity in provincial-level regions and the GTFP of registered manufacturing enterprises in the province may be because regions with lower energy intensity have more efficient resource utilization and cost advantages, which enhances the productivity and competitiveness of enterprises.

### 5.2 Robustness and endogeneity tests

To ensure the accuracy and robustness of the regression results, several robustness and endogeneity tests were conducted. First, to address the potential influence of outliers on the regression results, all variables are trimmed at the 1 % level on both sides. The results are shown in Column (1) of Table 3. Second, the measure of the dependent variable was replaced, and the directional distance function was used to calculate GTFP for regression, as shown in Column (2). Third, a first-order difference (FD) test was also conducted, and the results are presented in Column (3). These robustness tests show that the signs and significance of each explanatory variable remains significant. This indicates that the positive impact of manufacturing service orientation on GTFP is robust.

**Table 3:** Results of the robustness and endogeneity tests.

Variables	(1)	(2)	(3)	(4)	(5)
	Tail contraction	Recalculating GTFP	FD	IV-2SLS	
lnSer		0.610* (0.359)			
W.lnSer	1.166** (0.582)				
D1.lnSer			2.262** (0.838)		
L.lnSer				0.296* (0.170)	
lnSerprop					2.020** (0.813)
N	106	110	36	74	106
R^2^	0.628	0.560	0.490	0.860	0.723
Fixed effect	Yes	Yes	Yes	Yes	Yes
Control variables	Yes	Yes	Yes	Yes	Yes
Kleibergen‒Paap rk LM statistic				14.390***	4.385**
Kleibergen‒Paap rk Wald F statistic				21.596*	16.736*

Note: *, **, and *** represent significance at the 10 %, 5 %, and 1 % levels, respectively. Robust standard errors are displayed in parentheses. The regression results for control variables are omitted but are available upon request.

To address the potential endogeneity issues arising from self-selection bias, reverse causality, and omitted variables, two instrumental variables were employed in a two-stage least squares regression (IV-2SLS). First, lagged manufacturing output servitization (L.lnSer) was used as an instrument for lnSer. The results are presented in Column (4) of Table 3. Second, we utilized the share of the service sector in GDP at the company's registered location (lnSerprop) as an instrument for lnSer, and the results can be found in Column (5). The development of the service sector in the company's registered location can influence the level of servitization within local manufacturing companies, but it is less likely to be affected by the servitization of individual manufacturing firms. The results of the underidentification test and weak identification test both indicate that the selected instrumental variables are appropriate. These results reject the influence of endogeneity, verifying that the conclusion of this paper remains robust, thus further supporting the positive impact of manufacturing service orientation on GTFP.

### 5.3 Mediation effect test

To further explore the mechanism behind the positive impact of manufacturing output servitization on GTFP, the potential mediating effects of profitability and R&D capability are examined in this section. Specifically, enterprise profit margin (Pro) is used to measure profitability, while R&D investment ratio (Inn) is used to measure R&D capability. Furthermore, the Sobel test is used to examine the mediating effect.

The results of using profit margin as a mediator variable are presented in Columns (1) and (2) of Table 4. In the first stage, manufacturing output servitization is positively correlated with enterprise profit margin. By leveraging digital technology and intelligent equipment, manufacturing output servitization optimizes and automates the production process, leading to reduced production costs. This, in turn, enhances production efficiency, shortens the production cycle, accelerates product launch, and improves market competitiveness. Additionally, manufacturing output servitization combines product sales with services to provide more comprehensive solutions, thus increasing product value-added and improve product prices and profit margins. In the second stage, enterprise profit margin is positively correlated with GTFP. A higher net profit margin of total assets indicates stronger profitability, which means that more funds can be allocated toward the investment and innovation in green production. Companies can improve their GTFP by adopting more environmentally friendly technologies and materials, enhancing their production processes, and improving their product design. Although these investments and innovations may require additional costs, higher profit margins make it easier for companies to bear these costs to obtain greater returns. In summary, the findings support H2, which suggests that profitability serves as a mediator in the positive relationship between manufacturing service orientation and GTFP.

**Table 4:** Regression results of the mediating effect.

Variables	(1) Pro	(2) lnGTFP	(3) Inn	(4) lnGTFP
lnSer	0.055** (0.025)	0.709* (0.420)	0.333* (0.191)	1.034** (0.485)
Pro		0.470** (0.284)		
Inn				0.002* (0.001)
N	212	106	199	106
R^2^	0.874	0.801	0.989	0.760
Fixed effect	Yes	Yes	Yes	Yes
Control variables	Yes	Yes	Yes	Yes
Sobel	0.065* (0.037)		0.026* (0.015)	

Note: *, **, and *** represent significance at the 10 %, 5 % and 1 % levels, respectively. Robust standard errors are displayed in parentheses. The regression results for control variables are omitted but available upon request.

The results of using R&D investment capability as a mediator variable are presented in Columns (3) and (4) of Table 4. In the first stage, it is observed that manufacturing output servitization is positively correlated with the R&D investment ratio. By promoting technological innovation and knowledge accumulation, manufacturing output servitization enables companies to learn advanced technologies and management experience, thereby improving their technological level and innovation ability. Additionally, manufacturing output servitization can help companies build a more comprehensive knowledge management system, strengthen their knowledge management capacity, and increase their R&D capability. In the second stage, it can be seen that the R&D investment ratio is positively correlated with GTFP. First, to achieve green production, improvements and upgrades in technology and management are needed in the areas of product design, production processes, material selection, energy use, and more. By enhancing technological innovation capabilities and management practices, companies can better grasp relevant technologies and methods, enabling more effective implementation of green production. Furthermore, improving innovation capabilities also helps companies strengthen environmental and resource management and utilization, allowing for the formulation and implementation of more scientific, rational, and effective green production plans. Lastly, with increasing consumer demands for environmental protection and sustainable development, companies that excel in product innovation, environmental protection, social responsibility, and other aspects through enhanced innovation capabilities will be more attractive, gain a larger market share, and further improve GTFP. In summary, these findings support H3, which suggests that the R&D capability serves as a mediator in the positive relationship between manufacturing output servitization and GTFP.

### 5.4 Heterogeneity analysis

It is important to consider the heterogeneity of both the enterprise and its geographic location. Dividing manufacturing enterprises into developed and less developed regions is necessary because there may be differences in the level of economic development, technological advancement, and environmental regulations between these region types. By separately analyzing the impact of manufacturing output servitization on GTFP for each of these regions, we can gain a more nuanced understanding of the relationship between the two variables and identify any potential regional differences. We use the eastern coastal provinces of China (excluding Hong Kong, Macao and Taiwan) with a per capita GDP higher than the national average as developed regions, and provinces outside the coastal region with a per capita GDP lower than the national average as underdeveloped regions. This division is widely used in academic research and policy analysis in China and has been found to be a useful tool for understanding the regional differences in economic and social development.

As shown in Table 5 (1) and (2), regional heterogeneity plays a significant role in the impact of manufacturing output servitization on GTFP, as the level of manufacturing development and market environment varies between developed and less developed regions. These results indicate that the impact of output servitization on the GTFP of manufacturing enterprises in developed regions is smaller than that in less developed regions. This can be attributed to several factors. First, developed regions have a larger market scale, and the service industry is relatively mature. As a result, more capital and talent are attracted to the service industry, which may reduce the level of investment in environmental protection technology and energy conservation and the level of emission reduction achieved in the manufacturing industry, thereby inhibiting the improvement of GTFP. In contrast, the manufacturing industry still dominates the service industry in less developed regions, and the market environment is not as mature as that in developed regions. In the process of manufacturing output servitization, enterprises in less developed regions face more severe competitive pressure and resource constraints. Therefore, they may pay more attention to the application of energy conservation, emission reduction, and environmental protection technology, thereby increasing GTFP. Additionally, the relevant environmental policies and regulations in developed regions may be more inclusive and flexible, as such regions are an important economic development policy area. This may lead to a less urgent need for companies to invest in environmental protection technology, energy conservation and emission reduction, as companies can rely on government policies and regulations to meet environmental standards. In summary, this heterogeneity analysis highlights the importance of considering regional differences when exploring the impact of manufacturing output servitization on GTFP.

**Table 5:** Group regression results.

	(1) Developed area	(2) Less developed regions	(3) State-owned enterprises	(4) Foreign and private enterprises	（5） Non-GRI	（6） GRI
lnSer	0.957* (0.543)	4.612*** (1.217)	−0.149 (0.911)	1.249** (0.588)	0.377** (0.162)	3.529*** (0.888)
N	73	33	50	56	55	51
R^2^	0.758	0.632	0.934	0.800	0.827	0.799
Fixed effect	Yes	Yes	Yes	Yes	Yes	Yes
Control variables	Yes	Yes	Yes	Yes	Yes	Yes
Chow test	0.293* (0.168)		−0.081*** (0.014)		0.067* (0.039)	

Note: *, **, and *** represent significance at the 10 %, 5 % and 1 % levels, respectively. Robust standard errors are displayed in parentheses. The omitted regression results of controlled variables are available upon request.

In addition to geographical division, dividing manufacturing enterprises into nonstate-owned (foreign and private) and state-owned enterprises furthers our understanding of the impact of manufacturing output servitization on GTFP. State-owned enterprises often have different management structures, decision-making processes, and incentives than non-state-owned enterprises, which can affect the implementation and effectiveness of servitization strategies. The basis of this division can be clarified as arising from ownership structure and control. Non-state-owned enterprises are either privately or foreign-owned, with ownership and control concentrated in private hands, while state-owned enterprises are government-owned and operated, with their ownership and control vested in the state.

Table 5 (3) and (4) reveal that the impact of SOM on GTFP in state-owned enterprises is not significant. First, state-owned enterprises typically have relatively low efficiency and innovation, and their business models and management methods are prone to ossification. Even if they undergo manufacturing output servitization, it may be difficult to achieve truly efficient production and technological advancement. Second, GTFP refers to improving production efficiency and reducing costs while protecting the environment and resources, which involves improvements and upgrades in technology, management, and other aspects. State-owned enterprises may be less sensitive to the pressure of market competition, leading to a relatively lower willingness to carry out necessary technological and management innovation. Finally, one of the main expressions of servitization in some state-owned manufacturing enterprises is to outsource some of their service links to professional service companies. However, these links often are not focused on issues such as the utilization efficiency of the environment and resources. Therefore, even if state-owned manufacturing enterprises carry out outsourcing-style service-oriented transformation, it cannot guarantee the effective improvement of GTFP. Furthermore, state-owned manufacturing enterprises tend to have high capital intensity, which might be due to factors such as government ownership and investment policies that prioritize large-scale infrastructure and equipment. However, high capital intensity may also inhibit the positive effect of output servitization on GTFP, as these enterprises may have limited flexibility to adapt to new business models that involve the provision of services. In summary, the analysis suggests that state-owned enterprises, due to their unique characteristics and limitations, may face more challenges in achieving GTFP through the mechanism of manufacturing output servitization.

Finally, this section compared two groups of companies: those that reference the Global Reporting Initiative (GRI) framework and those that do not. The research results, as shown in Table 5 (5) and (6), indicate that the positive impact of manufacturing output servitization on GTFP is greater for companies that reference GRI compared to those that do not. This could be due to the fact that GRI reporting encourages companies to consider their overall impact on society and the environment, leading to more sustainable and efficient business practices. It also indicates that there may be additional benefits to integrating ESG considerations into the servitization process.

## 6 Conclusion

### 6.1 Discussion

The findings of this study contribute to the existing literature by providing empirical evidence on the impact of manufacturing output servitization on GTFP in the context of China. The results confirm that manufacturing output servitization can directly enhance GTFP by improving the efficiency and environmental performance of the production process. This aligns with previous studies that have highlighted the positive relationship between servitization and environmental sustainability [47].

Moreover, the study underscores the importance of profitability and innovation capabilities in improving GTFP through manufacturing output servitization. By integrating services into their offerings, companies can not only generate additional revenue streams but also foster innovation and develop more sustainable production methods. These findings support the notion that servitization can be a strategic approach for companies to achieve economic growth while reducing their environmental footprint [50].

The differential effect of manufacturing output servitization on GTFP between less developed and developed regions is an interesting finding. The greater positive impact observed in less developed regions suggests that servitization practices can play a crucial role in enhancing economic efficiency and environmental sustainability in these areas. This implies that policymakers and business leaders should prioritize targeted support and incentives for manufacturing companies in less developed regions to adopt servitization strategies. This can help bridge the regional development gap and promote sustainable industrial growth [51].

The varying impact of manufacturing output servitization on GTFP among different types of enterprises is another important finding. Private and foreign-owned enterprises experience a more significant positive effect on GTFP compared to state-owned enterprises. This highlights the potential of servitization as a strategy for private and foreign-funded companies to improve both their economic and environmental performance [52]. It also suggests that state-owned enterprises may require additional support or specific policy measures to fully leverage the benefits of servitization. The research also highlights the role of ESG reporting frameworks, specifically the GRI, in enhancing the positive impact of manufacturing output servitization on GTFP. Companies that adhere to the GRI framework and reflect their ESG performance are more likely to experience a greater improvement in GTFP through servitization efforts. This emphasizes the importance of transparent and accountable reporting practices in driving sustainable manufacturing practices [53].

This study contributes to the field of sustainable manufacturing by demonstrating the positive impact of manufacturing output servitization on GTFP in the context of China, thereby advancing our understanding of the mechanisms through which manufacturing practices can be aligned with sustainability goals. It provides empirical evidence of the potential of servitization to promote environmentally friendly manufacturing processes and enhance overall economic sustainability. Additionally, the research bridges a gap in the existing literature by focusing on the unique dynamics and outcomes of servitization within the Chinese manufacturing landscape. The practical implications of the study's findings extend to other countries and regions seeking to balance economic development and environmental protection. The results highlight the need for policymakers to create a supportive environment and develop policies that encourage companies to adopt servitization strategies. Furthermore, the study emphasizes the importance of industry stakeholders actively engaging in knowledge sharing and collaboration to identify and implement best practices for sustainable manufacturing.

### 6.2 Suggestion

Promoting service transformation and improving GTFP are vital directions for manufacturing enterprises seeking to advance their transformation and upgrading efforts while contributing to green, low-carbon and high-quality development. For example, Lăzăroiu et al. [54] explored the application of artificial intelligence-based decision-making algorithms and big data-driven cognitive manufacturing in sustainable cyber-physical management systems. This study is particularly important in the current context regarding sustainable development, particularly that based on the integration of intelligent services and information services in the manufacturing industry. This paper offers several suggestions for achieving this goal:

Manufacturing companies are encouraged to actively pursue servicizing their operations by integrating services into their offerings. This may involve providing after-sales services, maintenance and repair services, or offering value-added services such as customization or consulting. By doing so, companies can enhance their GTFP by making their production process more efficient and environmentally friendly. Additionally, it is crucial for companies to prioritize profitability and innovation capabilities, as these factors play a significant role in improving GTFP. Therefore, investing in research and development activities to foster innovation and adopting sustainable and eco-friendly production methods can be beneficial. Moreover, policymakers and business leaders should pay special attention to less developed regions, as the positive effect of service-oriented transformation on GTFP is greater in these areas compared to developed regions. Targeted support and incentives for companies in less developed regions to adopt manufacturing servitization practices, including financial support, technological assistance, and regulatory measures, can be instrumental. Lastly, a collaborative approach between policymakers, industry stakeholders, and academia is necessary to promote sustainable manufacturing practices. Creating a conducive environment and supportive policies that encourage companies to adopt servitization strategies, as well as active participation in knowledge sharing and collaboration to identify and implement best practices for sustainable manufacturing, are essential for achieving environmental, social, and economic goals.

### 6.3 Limitations and prospects

Despite the valuable insights gained from this study, it is important to acknowledge the limitations stemming from the sample size and unbalanced panel data. The small and unbalanced nature of the dataset, particularly in regard to carbon emissions and other relevant data from Chinese manufacturing listed companies, may restrict the generalizability of the findings and their applicability to a broader context. These limitations should be considered when interpreting the results and applying them to other industries or countries.

Moving forward, future research could aim to address the limitations by seeking access to a larger and more balanced dataset. This would enable a more comprehensive and representative analysis of the impact of manufacturing output servitization on GTFP and its implications for sustainable practices. Additionally, exploring the potential influence of additional variables and sustainability indicators, such as specific environmental metrics, could provide a more nuanced understanding of the relationship between servitization and sustainable economic efficiency. It is also possible to consider discussing the positive relationship between the manufacturing output servitization and GTFP from the perspectives of digital technology, informatization, and public environmental awareness, in order to explore additional enlightening or underlying mechanisms. These efforts would contribute to a more robust and holistic assessment, offering valuable insights for policymakers and industry leaders seeking to advance sustainable manufacturing practices.

## Data availability

The data used in the paper can be found at the following link: https://doi.org/10.13140/RG.2.2.27529.62562.

## CRediT authorship contribution statement

**Hongsen Wang:** Writing – original draft, Methodology, Data curation. **Martin Lockett:** Writing – review & editing, Supervision. **Dongni He:** Funding acquisition, Formal analysis. **Yiqing Lv:** Software, Conceptualization.

## Declaration of competing interest

The authors declare the following financial interests/personal relationships which may be considered as potential competing interests:Hongsen Wang reports financial support was provided by 10.13039/501100012325National Office for Philosophy and Social Sciences of China.
